# Using infrared thermography to determine changes in teat skin surface temperature after machine milking in dairy cows

**DOI:** 10.3168/jdsc.2023-0542

**Published:** 2024-03-02

**Authors:** L.A. Harper, C. DiLeo, P.S. Basran, M. Wieland

**Affiliations:** 1 College of Veterinary Medicine, Cornell University, Ithaca, NY 14853; 2 Department of Clinical Sciences, College of Veterinary Medicine, Cornell University, Ithaca, NY 14853; 3 Department of Population Medicine and Diagnostic Sciences, College of Veterinary Medicine, Cornell University, Ithaca, NY 14853

## Abstract

Mastitis is among the costliest diseases affecting dairy cows, partly due to the resulting permanent reduction in the quantity and quality of milk produced. Most mastitis cases involve pathogenic organisms entering the cow's mammary gland through the teat canal. The teat has natural defenses against these pathogens that can be disrupted during milk harvesting. These disruptions of the teat's circulatory system and tissue integrity can predispose them to mastitis. Traditionally, machine milking–induced changes in teat blood circulation and tissue integrity have been assessed by means of manual evaluation and ultrasonography. Infrared thermography has previously been shown to produce precise and consistent measurements of skin surface temperatures (SST) on cows' hind teats. Our objective was to describe the variability in the teat SST following machine milking. Describing the variability in teat SST before and after milking could be useful to guide further studies to elucidate the physiology of the effects of milking on teat defense mechanisms. In this observational study, thermographic images of both hind teats from 140 cows immediately pre- and postmachine milking were analyzed. The average SST were subsequently determined at the proximal, middle, and distal aspects of each hind teat using image analysis software. The LSM (95% CI) from general linear mixed models of the pre- and postmilking SST, respectively, were 33.6 (33.5–33.8)°C and 35.4 (35.3–35.5)°C at the proximal aspect, 33.2 (33.1–33.4)°C and 35.2 (35.1–35.3)°C at the middle aspect, and 32.3 (32.1–32.5)°C and 34.0 (33.9–34.1)°C at the distal aspect. The observed increase in SST from pre- to postmilking SST at all 3 aspects of the teat suggest that some of the variability in the SST can be attributed to the milking event. Future research is warranted to investigate the biological relevance of SST changes during machine milking and any potential change in teat defense mechanisms, risk of mastitis, or other pathologies.

## Highlights

• Infrared thermography can measure skin surface temperature.
• Teat skin surface temperatures were obtained from the hind teats before and after machine milking.
• Teat skin surface temperatures increased at the proximal, middle, and distal teat aspects postmilking relative to premilking unit attachment.

## Graphical Abstract

**Figure:**
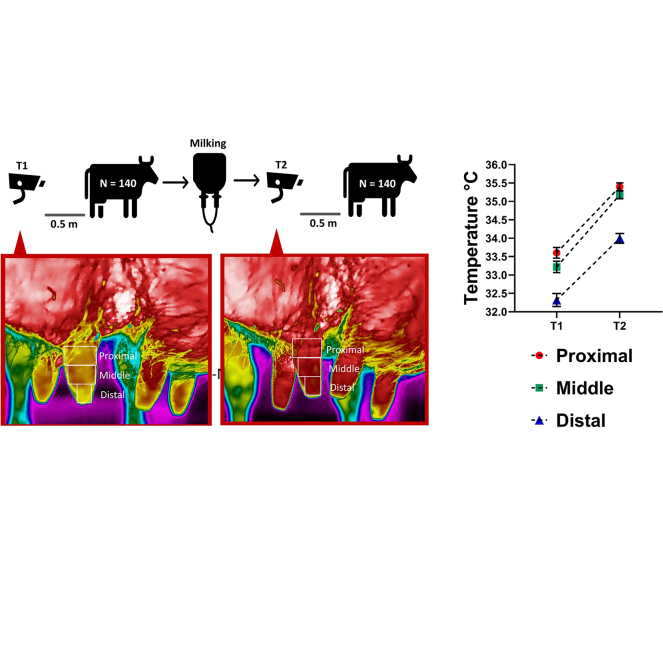
**Summary:** Thermographic images of both hind teats from 140 cows were obtained immediately before (T1) and after (T2) machine milking. Subsequently, the average teat skin surface temperatures at the proximal, middle, and distal aspects of the teat were determined with image analysis software. The average teat skin surface temperatures at the proximal, middle, and distal aspects of the teat were calculated using generalized linear mixed models. Least squares means (95% confidence intervals) of the pre- and postmilking values, respectively, were 33.6 (33.5-33.8)°C and 35.4 (35.3-35.5)°C at the proximal aspect, 33.2 (33.1-33.4)°C and 35.2 (35.1-35.3)°C at the middle aspect, and 32.3 (32.1-32.5)°C and 34.0 (33.9-34.1)°C at the distal aspect.

Mastitis, or the inflammation of the mammary gland, can result in a permanent decrease in milk quantity and quality, an increase in culling rates (animal loss costs), and an increase in treatment-related expenses. This, in addition to its prevalence, is why mastitis is among the most expensive diseases affecting the dairy industry. Most cases of mastitis are initiated by a pathogenic organism migrating into the mammary gland through the associated teat canal (Erskine, 2020). The cow's teat has natural defenses against pathogen migration, such as hydrophobic lipids, a continuously shedding, keratinized epithelial lining of the teat canal, healthy microbiota, and epithelial cell phagocytosis of inflammatory mediators (Katsafadou et al., 2019). However, an excessive vacuum, particularly during low milk flow, can result in excessive sloughing off of these natural defenses. Therefore, the milking procedure plays an active role in the prevention of, or predisposition of, cows to mastitis, and an effort should be made to protect the teat canal and associated tissues during all milking events. However, methods must exist on farm that distinguish healthy from compromised teat tissue associated with milking to ensure protection and proper milking parameters.

One method that can be implemented is monitoring teats for short-term changes (**STC**), which are teat tissue changes associated with the milking procedure, such as change in morphology and color (Mir et al., 2015). Changes in skin surface color could suggest poor perfusion of the tissue, which can result in hypoxia at the teat end (Mein, 2012). Hypoxia can lead to necrosis of the teat epithelial tissue, which weakens the first-line defenses of the mammary gland against mastitis. Other STC include firmness, thickening, or a ring at the teat base, and the degree of opening at the teat orifice, which are associated with harmful practices such as over-milking, poor pulsation, or an excessive vacuum (Hillerton et al., 2000). In addition to its detrimental effect on udder health, these STC have been reported to diminish animal well-being (Hillerton et al., 2002). Overmilking has been shown to lead to increased mastitis infections in unaffected quarters of infected cows during low milk flow (Natzke et al., 1982). Petersen (1944) also showed that when the teat cup creeps upward on the teat, it can cut off milk flow before the teat sinus, which can damage the membranes of the teat, cause ischemic injury, and predispose the cow to an increased risk of mastitis. Monitoring for these STC could allow for the detection of inappropriate machine milking protocols, provide an indirect measure of the teats' defense mechanisms, and offer opportunities for correction to decrease the risks associated with mastitis development.

Ideally, every teat of every milked cow would receive individualized care and machine milking protocols to minimize the risk of mastitis. Each teat should be monitored at every milking for changes that could predispose the associated mammary gland to mastitis development. However, there are currently over 9.4 million dairy cows in the United States and only approximately 106,000 individuals employed throughout all of dairy manufacturing (IBISWorld, 2024). Shifting staff to monitor for STC would be costly and time consuming and likely outweigh the benefits of individualized monitoring. However, an automated monitoring procedure could reap the benefits of individualized teat care without incurring the costs associated with increased technician time and salary.

Digital imaging is capable of detecting STC such as color changes, the presence of a ring, and teat canal openness but is limited in its ability to determine teat-end firmness due to congestion, which historically has been detected through manual palpation. Infrared thermography (**IRT**) could potentially provide more information about the teat by also measuring teat skin surface temperature (**SST**). Previous IRT studies have been able to differentiate between the presence and absence of mastitis when comparing the SST of an infected udder with that of other quarters or of the body SST (Sathiyabarathi et al., 2016). Infrared thermography has also previously been shown to be useful in reflecting changes in the SST associated with different California Mastitis Test scores (Colak et al., 2008). The goal of this project was to describe changes in teat SST that occur during machine milking to eventually evaluate its use as a monitoring tool for STC and proper milking protocols.

This observational study was conducted at the Teaching Dairy Barn at Cornell University, College of Veterinary Medicine (Ithaca, NY) in July 2021. The study protocol was reviewed and approved by the Cornell University Institutional Animal Care and Use Committee (protocol no. 2021–0005). During the study period, approximately 160 lactating Holstein cows were housed in 2 freestall pens that were bedded with recycled sand. They were fed a TMR formulated to meet or exceed the requirements outlined by the NRC (2001). Herd data were maintained in a dairy management software program (Dairy Comp 305, Valley Agricultural Software, Tulare, CA). The farm used national DHIA services, including the individual-cow SCC option. The rolling herd key performance indicators were average milk production, 12,512 kg; bulk tank SCC, 225,000 cells/mL; monthly clinical mastitis incidence, 2.3%; 21-d pregnancy rate, 26%; and culling rate, 35.0%.

Cows were milked 3 times per day at 0400, 1100, and 1900 h in a double 10 parallel milking parlor (P2100, DeLaval International AB, Tumba, Sweden). The vacuum pump (7.5 kW) was set to supply a receiver operator vacuum of 45 kPa regulated by a variable frequency drive. The milking unit was composed of a cluster MC70 (DeLaval International AB) and a milking liner with a square barrel shape (ProSquare DPX2, IBA, Millbury, MA). The pulsators (EP100, DeLaval International AB) were set to a pulsation rate of 60 cycles/min, a ratio of 70:30, and a side-to-side alternating pulsation. These settings resulted in an average claw vacuum during the peak milk flow period of 37 kPa. The automatic cluster removers were set to a cluster remover milk flow threshold of 1.4 kg/min, a 0-s delay, and a vacuum decay time of 2.3 s. The milk sweep was initiated 1.5 s after unit retraction and lasted for 4 s. The milk line was installed 75 cm below the cow standing level. The milking parlor was equipped with electronic on-farm flow-through milk flow meters using near-infrared technology (MM27BC, DeLaval International AB) for the assessment of milking characteristics. The milking system settings and milking characteristics were monitored with a dairy farm management software program (DelPro, DeLaval International AB). Before the start of the study, all system settings were verified and assessed by the investigators according to the guidelines outlined by the National Mastitis Council (2012).

Lactating cows were eligible for enrollment if they were free of clinical mastitis for at least 2 wk, had no udder abnormalities such as nonlactating quarters or teat injuries, and had a record of normal ease of handling. We obtained cow characteristics such as lactation number, stage of lactation, and SCC on the last test day from the dairy management software program (DairyComp 305, Valley Agricultural Software, Tulare, CA). The milk flow characteristics were obtained with the electronic on-farm flow-through milk flow meters and recorded with the dairy farm management software (DelPro, DeLaval International AB): milk yield (i.e., yield of milk harvested from start of milking to detachment of the milking unit, kg), milking unit-on time (i.e., duration from start of milking to detachment of the milking unit, s), average milk flow rate (calculated as total/milking unit-on time, kg/min), and time spent in low milk flow rate (i.e., time spent below 1 kg/min milk flow rate between the start of milking and detachment of the milking unit, s).

To minimize interference with the dairy's milking routine, we collected the data during 3 routine milking sessions (2 milking sessions at 1100 h and 1 milking session at 1900 h). To facilitate data collection, one trained investigator (MW) performed the routine milking procedures, including premilking udder preparation, milking unit attachment, and application of a postmilking teat disinfectant. The premilking teat sanitization and stimulation consisted of 3 steps. Step 1 was to wipe all 4 teats with a clean cloth towel and dip the teats with an iodine-based teat dip (Multi Dose MD; DeLaval International AB); step 2 was to manually fore-strip and dry all 4 teats with a clean cloth towel; and step 3 was to attach and align the milking unit.

Thermographic images of both hind teats were obtained with a portable thermography camera (FLIR T530, Teledyne FLIR LLC, Wilsonville, OR) by 1 trained investigator (CD). Before the study, the camera was calibrated, and the imaging modes were identified and kept consistent throughout the trial. These included the autofocus function and the ‘laser' option, where the focus is based on a laser distance. The laser distance meter was enabled to automatically determine the object distance. The reflective temperature was kept at 20°C and the emissivity at 0.95. The atmospheric temperature and relative humidity were retrieved from the local weather station: d 1, 23°C, 81%; d 2, 24°C, 79%; and d 3, 23°C, 79%. Images were taken from the caudal aspect of the udder in a caudo-to-cranial direction from an approximately 0.5 m distance. Teat scans were taken after completion of premilking udder preparation before milking unit attachment (**T1**) and directly after unit detachment (**T2**). All scans were labeled with the cow identification number and the sequence using the note function and subsequently stored on the integrated flash drive.

One trained investigator (LH) conducted measurements of the teat SST of the left and right hind teats with the adjunct software program (FLIR Tools, Teledyne FLIR LLC) as previously described (DiLeo et al., 2022). Figure 1 depicts the regions of interest at the proximal, middle, and distal aspects of the teat used for the measurements of the average teat SST. We also assessed teat-end shape from the digital images, which were obtained automatically with the thermography camera and categorized them into pointed, round, and flat as previously described (Wieland et al., 2017). During the measurements, any notes, including information on cow identification number and sequence, remained obscured.

**Figure 1:**
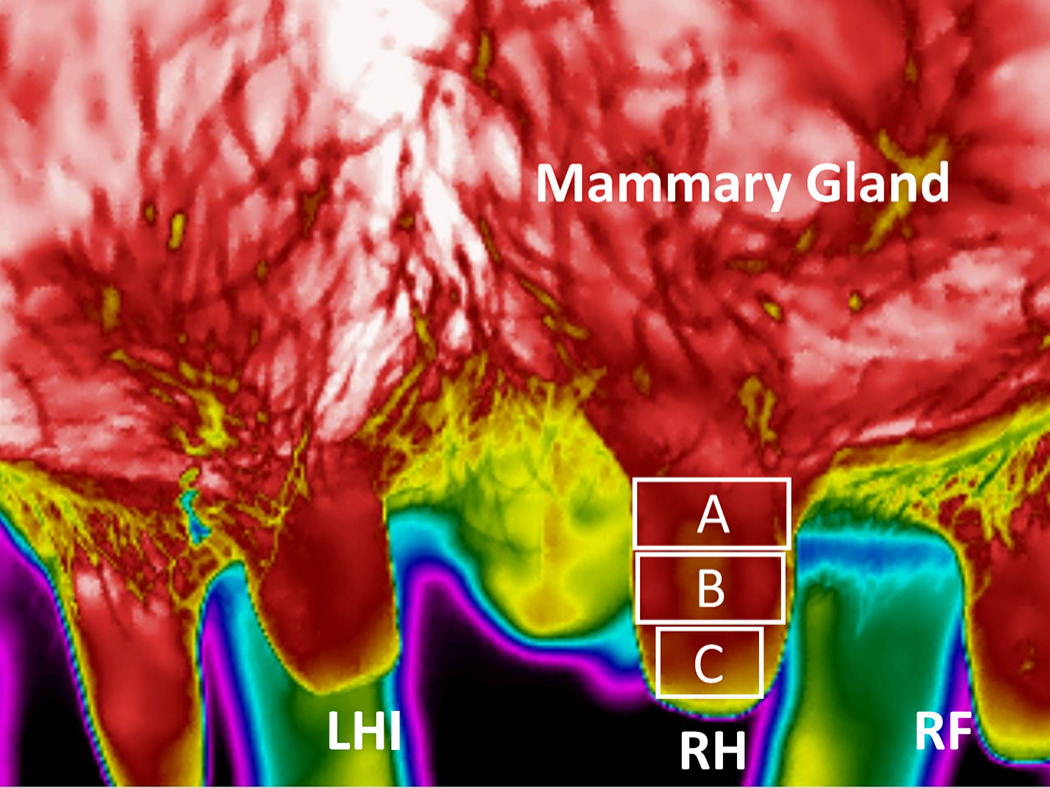
Thermographic image showing aspects of the mammary gland and the left hind (LHI), right front (RF), and right hind (RH) teats of a cow. The proximal (A), middle (B), and distal (C) aspects of the teat used to calculate the average teat skin surface temperatures of the 3 different regions of interest are depicted by the 3 rectangles overlaying the RH teat.

Data were compiled in Microsoft Excel (2019, Microsoft Corporation, Redmond, WA). Before the statistical analyses, we screened the data for missing values and removed observations with a missing teat scan or missing milk flow data. To describe the SST at the proximal, middle, and distal aspects of the teat relative to the machine milking event (i.e., T1 and T2), we fitted 3 general linear mixed models with PROC MIXED in SAS (version 9.4, SAS Institute Inc., Cary, NC). To account for the dependence of repeated measurements over time, a REPEATED statement for quarter position nested within cow was included. Four covariance structures were tested (compound symmetry, variance components, autoregressive order 1, and unstructured) to model the covariance of repeated measurements, and the one with the smallest Akaike's information criterion was selected. Time was forced into the models as a fixed effect. We considered lactation number (first, second, or ≥third lactation), stage of lactation (≤100, 101–200, or >200 DIM, SCC (log_10_-transformed), milk yield (kg/milking session), milking unit-on time (s), and teat-end shape as independent variables and initially screened them for inclusion with univariable analysis. All variables with *P* < 0.20 in this step were considered for inclusion in the initial models. We assessed collinearity among the eligible variables by calculating Spearman correlation coefficients in PROC CORR; a coefficient >|0.60| was considered indicative of collinearity. Manual backward elimination was used until each variable had *P* < 0.05 to achieve the final models. Finally, we calculated the LSM and 95% CI using the LSMEANS statement. Cook's distance was calculated with the INFLUENCE option to estimate the influence of outliers. A distance value of >0.5 was considered to indicate an influential value. For the final model, the assumptions of homoscedasticity and normality of residuals were assessed by inspecting residual plots versus corresponding predicted values and examining quantile—quantile residual plots.

A total of 147 cows were enrolled in the study, but data from 7 cows were excluded due to missing data (missing teat scan, n = 2; missing milk flow data, n = 5). The average (mean ± SD) DIM of the remaining 140 cows was 166 ± 117 d, ranging from 2 to 494 d. The lactation number was distributed as follows: 53 (37.9%) animals were in the first lactation, 35 (25.0%) animals were in the second lactation, and 52 (37.1%) animals were in their third or greater lactation (24 in the third; 12 in the fourth; 12 in the fifth; 3 in the sixth; and 1 in the seventh lactation). The median SCC was 57,000 cells/mL and ranged from 8,000 to 5,021,000. The average (mean ± SD [median; range]) values of the various milking characteristics from the studied milking observations were as follows: milk yield (12.7 ± 4.0 [12.3; 1.3–31.8] kg); average milk flow rate (3.1 ± 0.8 [3.0; 0.4–5.1] kg/min); milking unit-on time (247 ± 69 [240; 119–495] s); and time spent in low milk flow rate (21.4 ± 27.5 [14.9; 0–290] s). Teat-end shape was distributed as follows: pointed, 38 (13.6%); round, 203 (72.5%); and flat, 39 (13.9%). A total of 294 hind teats were studied using thermography at 2 time points (T1 and T2). Fourteen measurements were excluded corresponding to the 7 excluded cows. Thus, 280 teats were analyzed before (T1; 140 images) and after (T2; 140 images) machine milking. The average (mean ± SD) teat SST values at the proximal, middle, and distal aspects of the left and right hind teats are shown in Table 1.

**Table 1:** Average (mean ± SD) values of the teat skin surface temperature before (T1) and after (T2) machine milking at the proximal, middle, and distal aspects of the left and right hind teats of 140 Holstein dairy cows assessed with an infrared thermographic camera

Time point	Left hind teat			Right hind teat		
	Proximal	Middle	Distal	Proximal	Middle	Distal
T1 (°C)	33.6 ± 1.2	33.3 ± 1.3	32.4 ± 1.5	33.6 ± 1.2	33.2 ± 1.4	32.3 ± 1.6
T2 (°C)	35.4 ± 0.9	35.1 ± 0.9	34.0 ± 1.1	35.4 ± 0.9	35.2 ± 0.9	34.0 ± 1.1

For the proximal teat aspect, univariable analyses revealed the following results: lactation number, *P* = 0.15; stage of lactation, *P* = 0.11; logSCC, *P* = 0.02; milk yield, *P* = 0.0003; milking unit-on time, *P* = 0.11; and teat-end shape, *P* = 0.65. Spearman correlation coefficients indicated no collinearity among the 5 variables (r ≤ |0.53|), and thus, all 5 variables were included in the initial model. The final model included lactation number (*P* = 0.04), milk yield (*P* < 0.0001), and time of measurement (*P* < 0.0001). The LSM and 95% CI were 34.6°C (34.5–34.8) for cows in lactation 1, 34.5°C (34.3–34.7) for cows in lactation 2, and 34.3°C (34.2–34.5) for animals in lactation 3 and greater. A 1-unit increase in milk yield was associated with an increase of 0.05°C (95% CI, 0.03–0.08). The LSM and 95% CI were 33.6°C (33.5–33.7) at T1 and 35.4°C (35.3–35.5) at T2.

For the middle teat aspect univariable analyses revealed the following results: lactation number, *P* = 0.14; stage of lactation, *P* = 0.41, logSCC, *P* = 0.18; milk yield, *P* = 0.001; milking unit-on time, *P* = 0.08, and teat-end shape, *P* = 0.97. The final model included milk yield (*P* = 0.001) and time (*P* < 0.0001). A 1-unit increase in milk yield was associated with an increase of 0.04°C (95% CI, 0.02–0.07). The LSM and 95% CI were 33.2°C (33.1–33.4) at T1 and 35.2°C (35.1–35.3) at T2.

For the distal teat aspect univariable analyses revealed the following results: lactation number, lactation number, *P* = 0.49; stage of lactation, *P* = 0.93; logSCC, *P* = 0.17; milk yield, *P* = 0.71; milking unit-on time, *P* = 0.89; and teat-end shape, *P* = 0.44. The final model included time (*P* < 0.0001). The LSM and 95% CI were 32.3°C (32.1–32.5) at T1 and 34.0°C (33.9–34.1) at T2.

The inspection of Cook's distance revealed no influential outliers for any of the 3 final models. The assumptions of homoscedasticity and normality of residuals were met. Figure 2 shows the LSM and 95% CI for the SST at the proximal, middle, and distal aspects of the teat before (T1) and after (T2) machine milking.

**Figure 2:**
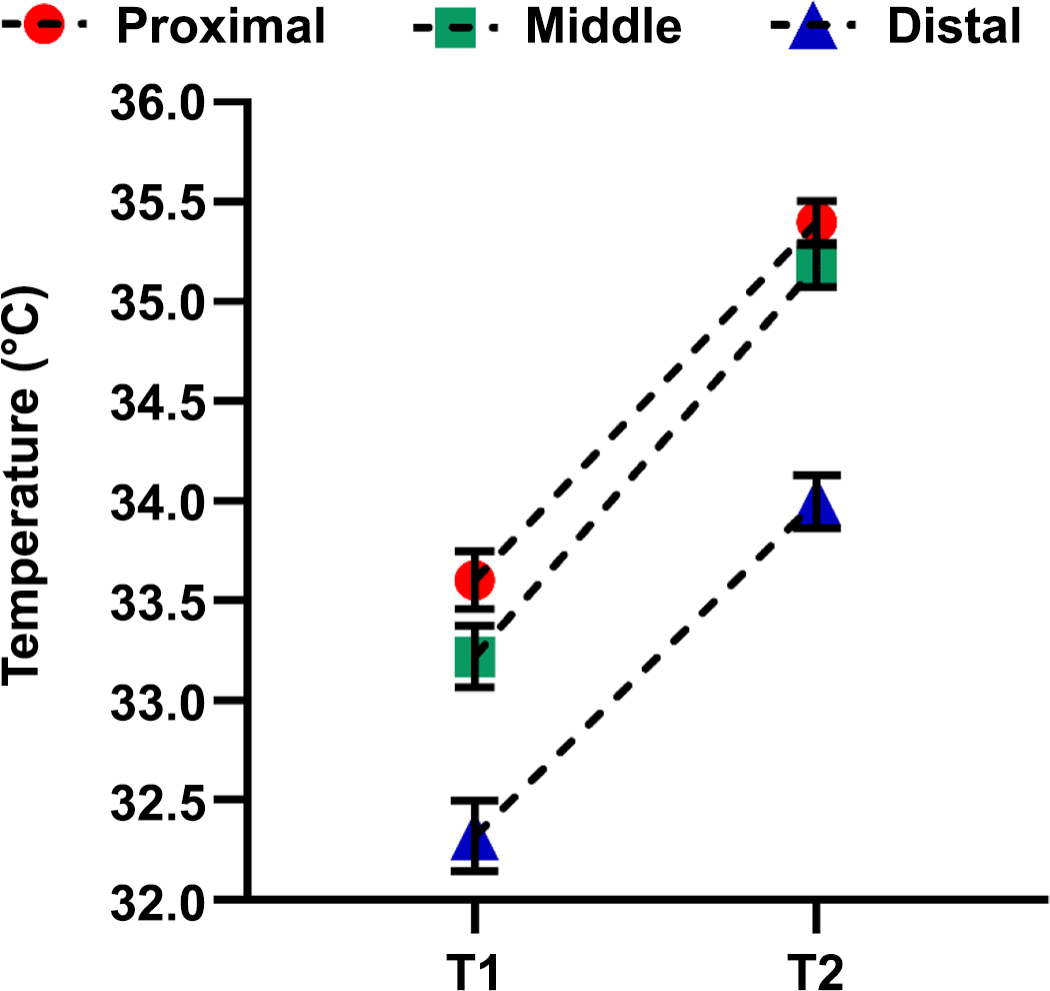
Least squares means from the general linear mixed models showing the skin surface temperatures at the proximal, middle, and distal aspects of the teat before (T1) and after (T2) machine milking as assessed with infrared thermography. Error bars show 95% CI.

Our objective was to describe changes in the SST using IRT that occur during the machine milking procedure. At both time points, the SST (determined by IRT) was higher proximally and cooler distally. This is contrary to the findings of Tangorra et al. (2019) but similar to those reported by Barth (2000). Differences in the study population and the assessment of the SST, including image capturing and the subsequent technique for measuring the average SST at the different teat aspects, could help explain the discrepancies among the studies. We observed that the SST increased at all 3 locations postmilking relative to the premilking value. The increase in the SST of the teats and quarters postmilking has been described previously (Paulrud et al., 2005; Vegricht et al., 2007; Yang et al., 2018; Barkova et al., 2019). Isaksson and Lind (1994) listed 3 reasons that could lead to increased SST postmilking: (1) warm milk flowing through the lumen of the teat, (2) insulation provided by the teat cup of the milking unit, and (3) physiologic responses in vascular plexuses. To demonstrate conductive heat transfer, they flushed 38°C to 39°C water through freshly slaughtered and excised teat cisterns and streak canals. They measured the temperature of the teat surface before and after flushing for less than 6 min with warm water and demonstrated an increase of 8°C (from 30°C to 38°C) in the teat surface temperature (Isaksson and Lind, 1994). Of course, this demonstration was on excised tissue and does not account for heat dissipation through the vascular system of the teat tissue back to the body. This dissipation of heat through the vascular system and through conduction to the teat cup, milking unit, and environment after milking unit detachment may account for the more minor rise in SST in our study than that of Isaksson and Lind (1994). In addition, postmilking, there is often a thin film of milk left on the teat that could dissipate heat through evaporation.

Isaksson and Lind (1994) proposed that milking induces relaxation, vasodilation, and an enhanced blood flow rate, which could increase the SST during and after milking. We previously demonstrated increased teat tissue perfusion after milking unit detachment compared with pre-stimulation through power Doppler sonography (Wieland et al., 2020). We also reported an increase in tissue perfusion post-teat preparation before milking; however, the study involved placing the teat into a warmed lube to scan with ultrasound, which could have relaxed the musculature in the tissue and simulated milking unit attachment, resulting in increased perfusion (Wieland et al., 2020). Increased teat tissue perfusion may be encouraged by the vacuum and a physiologic response to body-temperature milk flowing through the teat canal to prevent localized hyperthermia. Persson (1991) also found an increase in blood flow following machine milking at the teat end compared with 30 min before milking using laser Doppler flowmetry. Kunc et al. (2007) demonstrated that the teat SST increases after calf suckling, as well as after machine milking. Suckling is often considered a normal physiologic process, which may suggest that a rise in teat SST postmilking is not pathological. However, Neijenhuis et al. (2001) demonstrated that postmilking teat recovery could last longer than 8 h. Notably, teat-wall thickness took 6 h postmilking to return to baseline, which could be a proxy for vascular hyperemia to congestion or edema (Neijenhuis et al., 2001). This could suggest that the increased SST we observed postmilking may persist and merits further study in addition to determining the biological significance of prolonged teat tissue hyperemia.

Another factor that may have influenced the relative change in teat SST pre- and postmilking seen in our study is a relative decrease in SST following premilking teat preparation and stimulation. Isaksson and Lind's (1994) review of SST of teats associated with milking showed a repeatable decrease in SST following premilking teat stimulation. Our T1 followed premilking teat stimulation, cleaning with iodine, and fore-stripping. The manipulation of the teat end and dipping the teat in iodine likely resulted in a temporary physiological vasoconstriction that reduced heat dissipation through the vasculature and contraction of the teat, reducing skin surface area and further reducing heat loss. Similarly, because the iodine solution was not warmed, its impact on the SST was likely similar to the isopropyl alcohol challenge performed by Paulrud et al. (2005), in which teat SST decreased for up to 10 min after the exposure. Paulrud et al. (2005) and Hamann and Dück (1984) reported a decrease in temperature postmilking and dry or wet cleaning of the teat. This suggests that our T1 measurement may have been reduced following teat preparation, enhancing the change in temperature between T1 and T2.

Interestingly, teat-end shape was not associated with teat SST, which contrasts with previous findings from our group using B-mode ultrasonography (Wieland et al., 2019) and manual evaluation (Wieland et al., 2018) to assess changes in teat traits that occur relative to machine milking. We believe that the observed discrepancies can be attributed to differences in the study population and diagnostic techniques. Our study had some limitations. First, the study was conducted at a single farm with Holstein dairy cows that are milked 3 times per day. Thus, the external validity of the results is limited to similar operations in the region with the same milking schedule and similar milking machine settings (e.g., vacuum, pulsation, and take-off settings). Second, although we used a standard protocol for the assessment of the teat SST, the boundaries of the proximal, middle, and distal aspects may have changed slightly (between the pre- and postmilking images) due to, for example, changes in teat dimensions that occur during milking.

Throughout this work, we demonstrated repeatable increases in the teat SST at all teat aspects postmilking relative to premilking unit attachment and conclude that IRT can be used to reliably measure changes in teat SST that occur during machine milking. Future work should evaluate the biological significance of this change in temperature, determine how long it persists, and how it relates to machine milking. Such work could include more sophisticated methods such as thermal radiomics (Basran et al., 2022) and facilitate the evaluation of whether increased perfusion and temperature postmilking are pathological or protective against pathogens and intramammary infections.
